# Association of Physical Activity and Sitting Time Balance Index with all-cause and cause-specific mortality among cancer survivors in the USA: a cohort study

**DOI:** 10.1007/s00520-025-09709-x

**Published:** 2025-07-02

**Authors:** Yanxue Lian, Pincheng Luo

**Affiliations:** School of Medicine, University of Galway, Galway, Ireland

**Keywords:** Cancer survivors, Physical activity, Sedentary behavior, Mortality

## Abstract

**Purpose:**

This study examined the relationship between Physical Activity and Sitting Time Balance Index (PASTBI) and mortality outcomes, including all-cause, cardiovascular diseases (CVD)-, and cancer-specific mortality, in a nationally representative cohort of US cancer survivors.

**Methods:**

Data were derived from the National Health and Nutrition Examination Survey 2007–2018. The PASTBI was calculated using information from the Global Physical Activity Questionnaire, with mortality data obtained through linked databases. Cox proportional hazards models with survey sample weights were applied to estimate hazard ratios (HRs) for all-cause, CVD-related, and cancer-related mortality, adjusting for potential confounders.

**Results:**

The analysis included 3334 cancer survivors, with an average follow-up of 72.02 months. Compared with quartile 1, participants in quartile 4 (highest PASTBI) had significantly lower mortality risks, including 62% lower all-cause mortality [HR = 0.38; 95% CI = 0.29–0.50], 55% lower CVD mortality [HR = 0.45; 95% CI = 0.23–0.86], and 56% lower cancer mortality [HR = 0.44; 95% CI = 0.27–0.71]. Additionally, while both quartile 1 and quartile 2 reported no physical activity time, individuals in quartile 2, who had shorter sedentary time, exhibited lower mortality risks [all-cause mortality HR = 0.62; 95% CI = 0.49, 0.79; CVD mortality HR = 0.54; 95% CI = 0.35, 0.84; cancer mortality HR = 0.75; 95% CI = 0.54, 1.06].

**Conclusion:**

A higher PASTBI score, reflecting a better balance of physical activity and sedentary time, was associated with lower risks of all-cause, CVD-, and cancer-related mortality. Reducing sedentary time, even modestly, offers significant health benefits, independent of physical activity levels.

**Supplementary Information:**

The online version contains supplementary material available at 10.1007/s00520-025-09709-x.

## Introduction

Number of cancer survivors is rapidly increasing globally [1]. As of January 1, 2022, about 18.1 million adults were living with cancer in the USA [2], with an additional 2.0 million cases diagnosed annually [2]. This number is projected to continue to grow significantly, driven by advancements in early cancer detection, diagnosis, and treatment, and population aging [3]. By 2030, the number of cancer survivors in the USA is estimated to reach 21.6 million, rising further to 26 million by 2040 [4]. Despite these improvements in survival rates, many cancer survivors experience long-term and late effects from both the disease and its treatments [5], often resulting in reduced life expectancy [6]. Thus, identifying accessible and effective strategies to enhance the long-term health of cancer survivors has become an urgent public health priority.

Physical activity (PA) is a key lifestyle factor hypothesized to improve cancer survival through multiple mechanisms: (1) directly inhibiting tumor progression and metastasis, (2) increasing treatment efficacy, and (3) enhancing treatment completion rates [7]. Previous studies reported that physical activity, both before and after a cancer diagnosis, is linked to better survival outcomes [8], with stronger associations observed for post-diagnosis activity [9, 10]. Nevertheless, levels of physical activity among cancer survivors remain critically low, with more than one-third of this population engaging in minimal activity while spending prolonged periods in sedentary behaviors [11].

Sedentary behavior, characterized by waking activities that involve minimal energy expenditure (≤ 1.5 METs) in a sitting, reclining, or lying position, has become increasingly prevalent and is linked to numerous adverse health effects [12, 13]. Prolonged sedentary time in the general population has been associated with higher risks of cancer [14], CVD, diabetes [15], and all-cause mortality [16], particularly among those with insufficient physical activity [17]. Recognizing these risks, the 2020 World Health Organization Global Guidelines on Physical Activity and Sedentary Behavior emphasize reducing sedentary time and substituting it with physical activity to enhance health outcomes [18].

In cancer survivors, evidence regarding the combined impact of sedentary behavior and physical activity on mortality risk remains limited. Prior cohort studies have demonstrated that cancer survivors who are physically active have significantly lower risks of all-cause and cancer-specific mortality compared to those who are inactive [19]. Conversely, prolonged sitting (8 h or more per day) is strongly associated with higher mortality risks compared to sitting less than 4 h daily [19]. The combination of physical inactivity and prolonged sedentary time appears to result in the highest mortality risks in this population [19].

Interestingly, in populations without cancer, high levels of physical activity have been found to mitigate the detrimental effects of prolonged sedentary behavior, as highlighted in a harmonized meta-analysis [17]. This interplay between sedentary behavior and physical activity has prompted the creation of integrated measures, such as the Physical Activity and Sitting Time Balance Index (PASTBI), to simultaneously assess their impact on health outcomes. Botlero et al. demonstrated that an unfavorable balance between sedentary time and physical activity captured by PASTBI was strongly linked to increased mortality risk in adults with a mean age of 56.4 years [20].

Cancer survivors, however, tend to engage in more sedentary behavior compared to individuals without cancer [21, 22], largely due to comorbidities and physical deconditioning resulting from cancer and its treatments [23]. Despite this, limited research has explored the use of indices like PASTBI in assessing the combined effects of sedentary time and physical activity on survival in this population. Therefore, this study seeks to examine the relationship between PASTBI and mortality outcomes, including all-cause, cardiovascular diseases (CVD), and cancer-specific mortality, in a nationally representative cohort of US cancer survivors.

## Methods

### Study population

**Fig. 1:**
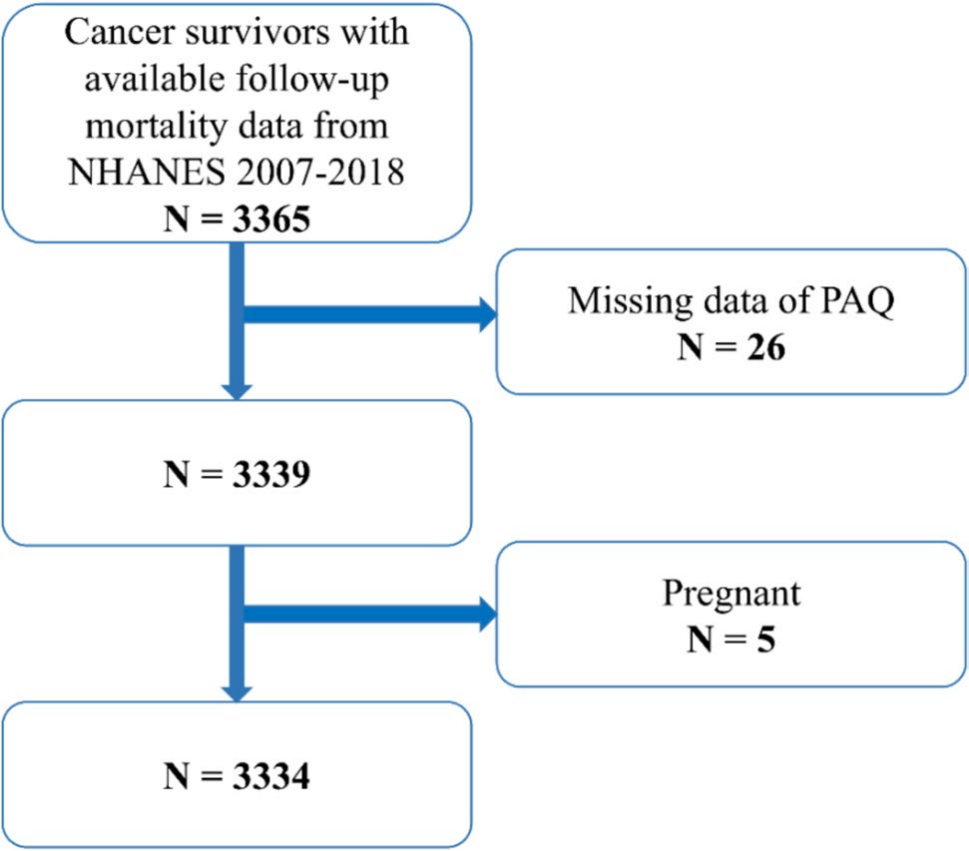
Flowchart of participant selection from NHANES 2007–2018. Abbreviations: NHANES, National Health and Nutrition Examination Survey; PAQ, Physical Activity Questionnaire

This study utilized data from the National Health and Nutrition Examination Survey (NHANES) (https://www.cdc.gov/nchs/nhanes/, accessed on December 10, 2024), a nationally representative survey approved by the National Center for Health Statistics (NCHS) Ethics Review Board. Written informed consent was obtained from all participants. Data from six survey cycles (2007–2008 to 2017–2018) were analyzed, focusing on cancer survivors. Cancer survivor status was determined based on a “yes” response to the question, “Has a doctor ever told you that you had other cancer?” Among 59,842 NHANES participants, 3365 were identified as cancer survivors with available follow-up mortality data. After excluding individuals with missing physical activity data (*n* = 26) and pregnant women (*n* = 5), the final sample comprised 3334 cancer survivors (Fig. 1).

### Assessment of physical activity, sedentary time, and PASTBI calculation

The Global Physical Activity Questionnaire was used to collect information on daily sedentary time and three main physical activity domains: occupational physical activity, transportation-related physical activity, and leisure time-related physical activity. It measured the frequency and duration of these activities over a typical week, with intensity assessed for occupational and leisure time activities. We focused our analysis on non-occupational physical activities as they represent modifiable behaviors under individual control, whereas occupational activities are constrained by employment requirements and their health benefits remain less well-established [24]. Drawing from prior research, non-occupational moderate-to-vigorous physical activity (MVPA) time (minutes/week) was computed by summing the time spent on transportation, moderate-intensity leisure time-related physical activity, and double the time spent on vigorous-intensity leisure time-related physical activities [20]. To address potential over-reporting, physical activity times exceeding 14 h (840 min) for any single activity type (*n* = 1) were recoded to 840 min [20].

Sedentary time was estimated by asking participants about the amount of time they typically spent sitting each day. This included activities such as sitting at home, at school, while traveling, or during leisure activities like reading or using a computer, but excluded sleep. Weekly sedentary time (hours/week) was calculated as follows:

$$\text{Weekly sedentary time (hours/week)}=\frac{\text{Daily sedentary time (minutes/day)}}{60}\times 7$$

To minimize reporting errors, sedentary times exceeding 18 h/day (126 h/week; *n* = 3) were recoded to 18 h/day [20]. For the calculation of the PASTBI, non-occupational MVPA or sedentary time values of zero were replaced with 0.1 min (non-occupational MVPA: *n* = 1703; sedentary time: *n* = 0) [20]. PASTBI was calculated using the formula:

$$\text{PASTBI}=\frac{\text{Weekly non-occupational MVPA time (minutes/week)}}{\text{Total weekly sedentary time (hours/week)}}$$

Higher PASTBI values represent a greater proportion of time spent engaging in non-occupational MVPA relative to sedentary behavior, whereas lower values indicate more sedentary time and less non-occupational MVPA.

### Mortality ascertainment

Mortality data were sourced from the NHANES public-use linked mortality file, which was updated through December 31, 2019. This file provides information on mortality status and underlying causes of death, classified according to the 10th revision of the International Classification of Diseases. The NCHS conducted the data linkage to the National Death Index using a probability-matching algorithm [25]. Detailed information on the matching methodology can be accessed through the NCHS [25]. This study primarily focused on all-cause mortality and deaths attributed to malignant neoplasms and heart disease.

### Confounders

Confounding variables were measured and collected concurrently with physical activity and sedentary time, with consideration of their associations with physical activity, sedentary time, and mortality [20, 26, 27]. Socio-demographic information was self-reported and included sex, age, marital status, education level, race, and poverty-income ratio (PIR). PIR was categorized as follows: ≥ 3.00 (high income), 2.00–2.99 (middle income), 1.00–1.99 (near poor), and < 1.00 (poor) [28].

Lifestyle factors included body mass index (BMI), classified according to standard categories [29]. Smoking status was categorized into three groups (current smokers, former smokers, and never smokers) based on the National Health Interview Survey Glossary [30]. Alcohol consumption was assessed with the following categories based on self-reported frequency of alcoholic beverage intake during the past 12 months: ≤ 1 drink, 2–14 drinks, ≥ 15 drinks, and missing data.

Chronic non-communicable diseases were identified through self-report, clinical examination, and laboratory data. These included hypertension (defined as a history of hypertension, use of antihypertensive medication, diastolic blood pressure ≥ 80 mmHg, or systolic blood pressure ≥ 130 mmHg) [31], diabetes (defined as a history of diabetes, use of hypoglycemic medications or insulin, fasting blood glucose ≥ 7.0 mmol/L, glycated hemoglobin ≥ 6.5%, or 2-h postprandial glucose ≥ 11.0 mmol/L) [32], and CVD, which were self-reported and included stroke, angina, coronary heart disease, heart attack, and congestive heart failure.

### Statistical analysis

To obtain representative estimates for the US population, adjustments for the complex survey design in NHANES data were applied, accounting for sample weights. Data analysis was conducted using R software (version 4.3.1; http://www.r-project.org, accessed on December 26, 2024) and EmpowerStats (version 4.2, http://www.empowerstats.com, accessed on December 26, 2024). Chi-square tests and independent *t*-tests were employed to compare categorical and continuous variables, respectively, between groups categorized by survival status (alive vs. deceased) and censoring status (uncensored vs. censored for CVD, and uncensored vs. censored for cancer). Chi-square tests and analysis of variance were used to examine differences in categorical and continuous variables, respectively, across quartiles of the PASTBI.

Continuous variables are presented as means ± standard deviation (SD), and while categorical variables are presented as frequencies (%). To examine the association of PASTBI with mortality outcomes, Cox proportional hazards models with survey sample weights were applied to estimate hazard ratios (HRs) for all-cause, CVD-, and cancer-related mortality, controlling for potential confounders. Three models were developed: model I (adjusted for socio-demographic), model II (additionally adjusted for lifestyle factors), model III (further adjusted for chronic non-communicable diseases). All statistical tests were two-tailed, with significance set at *P* < 0.05.

### Sensitivity analysis

To address potential reverse causality and test the robustness of our findings, we conducted several sensitivity analyses. First, we further adjusted for the time interval between cancer diagnosis and baseline measurement, in addition to the covariates included in model III. We then repeated the main analyses after excluding participants with less than 2 years of follow-up, those with underweight status (BMI < 18.5 kg/m^2^), and those with a history of CVD, including angina, coronary heart disease, congestive heart failure, heart attack, and stroke. Finally, we applied Fine-Gray competing risk models to account for competing events in cause-specific mortality analyses.

## Result

The analysis included 3334 cancer survivors, with an average age of 65.7 years. Baseline measurements were taken an average of 10.09 years (SD = 10.53) after cancer diagnosis, and participants were subsequently followed for an average of 72.02 months (SD = 42.01). Their baseline characteristics, stratified by quartiles of the PASTBI, are presented in Table 1. Compared to participants in quartile 1, characterized by lower non-occupational MVPA and higher sedentary behavior (lower PASTBI), those in quartile 4, who engaged in higher levels of non-occupational MVPA and less sedentary behavior (higher PASTBI), were more likely to be younger, male, of Mexican American or other Hispanic ethnicity, of other racial backgrounds, married or living with a partner, college-educated or higher, with higher incomes, normal BMI, no chronic diseases, lower alcohol consumption, and a history of never smoking.

**Table 1:** Baseline characteristics of cancer survivors by PASTBI quartile from NHANES 2007–2018

	Total (*N* = 3334)	Quartile 1 (*N* = 802)	Quartile 2 (*N* = 854)	Quartile 3 (*N* = 840)	Quartile 4 (*N* = 838)	*P*-value
Age (years)	65.7 ± 13.9	69.3 ± 12.3	66.9 ± 13.2	64.4 ± 13.6	62.4 ± 15.3	< 0.001
Age (years)						< 0.001
< 40	217 (6.5%)	26 (3.2%)	46 (5.39%)	51 (6.07%)	94 (11.22%)	
65 > age ≥ 40	1098 (32.9%)	220 (27.4%)	254 (29.74%)	320 (38.10%)	304 (36.28%)	
≥ 65	2019 (60.6%)	556 (69.3%)	554 (64.87%)	469 (55.83%)	440 (52.51%)	
Gender						< 0.001
Male	1586 (47.6%)	365 (45.5%)	356 (41.69%)	425 (50.60%)	440 (52.51%)	
Female	1748 (52.4%)	437 (54.5%)	498 (58.31%)	415 (49.40%)	398 (47.49%)	
Race						< 0.001
Mexican American	231 (6.9%)	32 (4.0%)	83 (9.72%)	51 (6.07%)	65 (7.76%)	
Other Hispanic	225 (6.7%)	40 (5.0%)	69 (8.08%)	46 (5.48%)	70 (8.35%)	
Non-Hispanic White	2189 (65.7%)	575 (71.7%)	516 (60.42%)	565 (67.26%)	533 (63.60%)	
Non-Hispanic Black	490 (14.7%)	112 (14.0%)	138 (16.16%)	130 (15.48%)	110 (13.13%)	
Other race	199 (6.0%)	43 (5.4%)	48 (5.62%)	48 (5.71%)	60 (7.16%)	
Marital status						< 0.001
Married	1852 (55.5%)	413 (51.5%)	489 (57.26%)	452 (53.81%)	498 (59.43%)	
Widowed	599 (18.0%)	189 (23.6%)	161 (18.85%)	141 (16.79%)	108 (12.89%)	
Divorced	445 (13.3%)	108 (13.5%)	106 (12.41%)	125 (14.88%)	106 (12.65%)	
Separated	105 (3.1%)	27 (3.4%)	23 (2.69%)	28 (3.33%)	27 (3.22%)	
Never married	223 (6.7%)	45 (5.6%)	50 (5.85%)	70 (8.33%)	58 (6.92%)	
Living with partner	110 (3.3%)	20 (2.5%)	25 (2.93%)	24 (2.86%)	41 (4.89%)	
Education						< 0.001
Less than 9th grade	309 (9.3%)	88 (11.0%)	113 (13.23%)	54 (6.43%)	54 (6.44%)	
9–11th grade	411 (12.3%)	126 (15.7%)	131 (15.34%)	82 (9.76%)	72 (8.59%)	
High school graduate	730 (21.9%)	168 (20.9%)	229 (26.81%)	176 (20.95%)	157 (18.74%)	
Some college or AA degree	1009 (30.3%)	254 (31.7%)	243 (28.45%)	250 (29.76%)	262 (31.26%)	
College graduate or above	875 (26.2%)	166 (20.7%)	138 (16.16%)	278 (33.10%)	293 (34.96%)	
PIR						< 0.001
< 1	479 (14.4%)	115 (14.3%)	150 (17.56%)	108 (12.86%)	106 (12.65%)	
2 > PIR ≥ 1	813 (24.4%)	231 (28.8%)	244 (28.57%)	181 (21.55%)	157 (18.74%)	
3 > PIR ≥ 2	802 (24.1%)	191 (23.8%)	228 (26.70%)	187 (22.26%)	196 (23.39%)	
≥ 3	1240 (37.2%)	265 (33.0%)	232 (27.17%)	364 (43.33%)	379 (45.23%)	
BMI (kg/m^2^)	29.1 ± 6.4	30.3 ± 7.1	29.2 ± 6.2	29.3 ± 6.7	27.8 ± 5.4	< 0.001
BMI (kg/m^2^)						< 0.001
< 18.5	50 (1.5%)	13 (1.6%)	16 (1.87%)	14 (1.67%)	7 (0.84%)	
25 > BMI ≥ 18.5	789 (23.7%)	131 (16.3%)	187 (21.90%)	208 (24.76%)	263 (31.38%)	
30 > BMI ≥ 25	1298 (38.9%)	316 (39.4%)	337 (39.46%)	308 (36.67%)	337 (40.21%)	
≥ 30	1197 (35.9%)	342 (42.6%)	314 (36.77%)	310 (36.90%)	231 (27.57%)	
Angina						< 0.001
Yes	174 (5.2%)	48 (6.0%)	63 (7.38%)	36 (4.29%)	27 (3.22%)	
No	3160 (94.8%)	754 (94.0%)	791 (92.62%)	804 (95.71%)	811 (96.78%)	
Coronary heart disease						< 0.001
Yes	317 (9.5%)	98 (12.2%)	91 (10.66%)	72 (8.57%)	56 (6.68%)	
No	3017 (90.5%)	704 (87.8%)	763 (89.34%)	768 (91.43%)	782 (93.32%)	
Congestive heart failure						< 0.001
Yes	235 (7.0%)	98 (12.2%)	71 (8.31%)	41 (4.88%)	25 (2.98%)	
No	3099 (93.0%)	704 (87.8%)	783 (91.69%)	799 (95.12%)	813 (97.02%)	
Diabetes						< 0.001
Yes	920 (27.6%)	267 (33.3%)	279 (32.67%)	223 (26.55%)	151 (18.02%)	
No	2414 (72.4%)	535 (66.7%)	575 (67.33%)	617 (73.45%)	687 (81.98%)	
Heart attack						< 0.001
Yes	327 (9.8%)	104 (13.0%)	98 (11.48%)	77 (9.17%)	48 (5.73%)	
No	3007 (90.2%)	698 (87.0%)	756 (88.52%)	763 (90.83%)	790 (94.27%)	
Hypertension						< 0.001
Yes	2591 (77.7%)	662 (82.5%)	697 (81.62%)	636 (75.71%)	596 (71.12%)	
No	743 (22.3%)	140 (17.5%)	157 (18.38%)	204 (24.29%)	242 (28.88%)	
Stroke						< 0.001
Yes	295 (8.8%)	106 (13.2%)	84 (9.84%)	55 (6.55%)	50 (5.97%)	
No	3039 (91.2%)	696 (86.8%)	770 (90.16%)	785 (93.45%)	788 (94.03%)	
Alcohol consumption						< 0.001
1	924 (27.7%)	196 (24.4%)	196 (22.95%)	282 (33.57%)	250 (29.83%)	
2–14	860 (25.8%)	171 (21.3%)	176 (20.61%)	230 (27.38%)	283 (33.77%)	
≥ 15	5 (0.1%)	1 (0.1%)	0 (0%)	1 (0.12%)	3 (0.36%)	
Missing	1545 (46.3%)	434 (54.1%)	482 (56.44%)	327 (38.93%)	302 (36.04%)	
Smoking status						< 0.001
Never smoker	1522 (45.7%)	325 (40.5%)	378 (44.26%)	424 (50.48%)	395 (47.14%)	
Former smoker	1290 (38.7%)	347 (43.3%)	306 (35.83%)	317 (37.74%)	320 (38.19%)	
Current smoker	522 (15.7%)	130 (16.2%)	170 (19.91%)	99 (11.79%)	123 (14.68%)	
Sedentary time (hours/weekly)	44.7 ± 23.0	68.2 ± 18.8	30.7 ± 9.4	48.8 ± 23.4	32.5 ± 17.3	< 0.001
Non-occupational MVPA (minutes/weekly)	184.7 ± 360.4	0.1 ± 0.00	0.10 ± 0.00	129.77 ± 95.60	604.62 ± 511.08	< 0.001
Months of follow-up	72.0 ± 42.0	60.6 ± 39.4	72.01 ± 44.36	76.78 ± 40.63	78.22 ± 41.21	< 0.001
Time from cancer diagnosis to baseline measurement (years)	10.09 ± 10.53	10.04 ± 11.33	10.09 ± 10.24	10.24 ± 10.73	9.99 ± 9.83	0.96
All-cause mortality						< 0.001
No	2485 (74.5%)	485 (60.5%)	605 (70.84%)	681 (81.07%)	714 (85.20%)	
Yes	849 (25.5%)	317 (39.5%)	249 (29.16%)	159 (18.93%)	124 (14.80%)	
Cancer mortality						< 0.001
No	3037 (91.09%)	704 (87.78%)	761 (89.11%)	784 (93.33%)	788 (94.03%)	
Yes	297 (8.91%)	98 (12.22%)	93 (10.89%)	56 (6.67%)	50 (5.97%)	
CVD mortality						< 0.001
No	3151 (94.51%)	733 (91.40%)	806 (94.38%)	800 (95.24%)	812 (96.90%)	
Yes	183 (5.49%)	69 (8.60%)	48 (5.64%)	40 (4.76%)	26 (3.10%)	

Continuous variables are presented as means ± standard deviation (SD), and while categorical variables are presented as frequencies (%)

*CVD*, cardiovascular diseases; *BMI*, body mass index; *PIR*, poverty-income ratio; *MVPA*, moderate-to-vigorous physical activity

During follow-up, 849 participants (25.5%) died from all causes, including 297 cancer-related deaths and 183 CVD-related deaths. The proportions of both all-cause and cause-specific mortality were lowest in quartile 4 and progressively increased with decreasing PASTBI scores, reflecting lower non-occupational MVPA and higher sedentary behavior. Surviving participants had significantly higher PASTBI scores than those who died from all causes during follow-up (Online Resource, Table S1). Similarly, in the analysis of CVD-related mortality, participants who were either alive or had non-CVD-related deaths (combined as the censored group) had significantly higher PASTBI scores than those who died from CVD-related causes. A comparable pattern emerged in the cancer-related mortality analysis, where individuals who were either alive or had non-cancer-related deaths (censored group) demonstrated significantly higher PASTBI scores than those who died from cancer.

Table 2 presents the results of the Cox proportional hazards regression models, illustrating the associations between the PASTBI and both all-cause and cause-specific mortality. Compared with participants in quartile 1 (lowest PASTBI), those in quartile 4, characterized by higher non-occupational MVPA and lower sedentary time, had significantly lower risks of both all-cause and cause-specific mortality. Specifically, quartile 4 was linked to a 62% reduction in all-cause mortality risk [0.38 (0.29, 0.50)], a 55% reduction in CVD-related mortality risk [0.45 (0.23, 0.86)], and a 56% reduction in cancer-related mortality risk [0.44 (0.27; 0.71)]. Additionally, while both quartile 1 and quartile 2 reported no non-occupational MVPA time, individuals in quartile 2, who had shorter sedentary time (sedentary time = 68.2 ± 18.8 h/week and 30.7 ± 9.4 in quartile 1 and quartile 2, respectively), exhibited lower mortality risks [all-cause mortality HR = 0.62 (0.49, 0.79); CVD mortality HR = 0.54 (0.35, 0.84); cancer mortality HR = 0.75 (0.54, 1.06)], with the reduction in cancer-related mortality not reaching statistical significance. Similar protective effects were observed for quartile 3 [all-cause mortality = 0.38 (0.30, 0.49); CVD-related mortality = 0.49 (0.30, 0.81); cancer-related mortality = 0.43 (0.28, 0.66)] compared to quartile 1. These findings were robust across multiple sensitivity analyses, including models with additional adjustment for the time interval between cancer diagnosis and baseline measurement (beyond the covariates in model III), exclusion of participants with short follow-up, underweight status, or CVD, and the use of competing risk models, all of which yielded consistent results (Online Resource, Tables S2–S6).

**Table 2:** Association of PASTBI with all-cause, CVD-, and cancer-related mortality

PASTBI quartiles	Person-years of follow-up	No. of events	Mortality rate (per 1000 person-y)	Adjusted HR (95%CI), *P*-value		
				Model I	Model II	Model III
All-cause mortality						
Quartile 1	385,022.9	317	0.82	Reference	Reference	Reference
Quartile 2	274,107.5	249	0.91	0.64 (0.51; 0.80)	0.62 (0.50; 0.78)	0.62 (0.49; 0.79)
Quartile 3	122,575.75	159	1.29	0.35 (0.28; 0.45)	0.37 (0.29; 0.47)	0.38 (0.30; 0.49)
Quartile 4	83,059.33	124	1.49	0.36 (0.27; 0.47)	0.35 (0.27, 0.46)	0.38 (0.29; 0.50)
* P* for trend				< 0.0001	< 0.0001	< 0.0001
CVD mortality						
Quartile 1	17,934.25	69	3.85	Reference	Reference	Reference
Quartile 2	11,568	48	4.14	0.56 (0.37; 0.85)	0.55 (0.36, 0.82)	0.54 (0.35; 0.84)
Quartile 3	7913.33	40	5.05	0.44 (0.27; 0.71)	0.46 (0.28, 0.75)	0.49 (0.30; 0.81)
Quartile 4	3605.33	26	7.21	0.36 (0.20; 0.65)	0.38 (0.20; 0.70)	0.45 (0.23; 0.86)
* P* for trend				< 0.0001	< 0.0001	< 0.05
Cancer mortality						
Quartile 1	31,939.83	98	3.07	Reference	Reference	Reference
Quartile 2	32,681.75	93	2.85	0.77 (0.54, 1.09)	0.75 (0.53; 1.05)	0.75 (0.54; 1.06)
Quartile 3	12,600	56	4.44	0.39 (0.26, 0.59)	0.42 (0.27; 0.64)	0.43 (0.28; 0.66)
Quartile 4	12,795.83	50	3.91	0.42 (0.27, 0.64)	0.42 (0.27; 0.67)	0.44 (0.27; 0.71)
* P* for trend				< 0.0001	< 0.0001	0.007

*PASTBI*, Physical Activity and Sitting Time Balance Index; *CVD*, cardiovascular diseases

Model I adjusted for age, gender, race, marital status, education level, and PIR

Model II additionally adjusted for BMI, smoking status, and alcohol consumption

Model II further adjusted for hypertension, diabetes, angina, coronary heart disease, heart attack, congestive heart failure, and stroke

## Discussion

The main finding of this study was that a more favorable balance between these two health behaviors, indicated by a higher PASTBI, was strongly linked to reduced risks of all-cause mortality, cancer-related mortality, and CVD-related mortality. This aligns with existing evidence emphasizing the joint impact of sedentary time and non-occupational MVPA on health outcomes. For example, Cao et al. (2022) reported that prolonged sitting was associated with higher mortality risks only among cancer survivors who were inactive or insufficiently active, whereas sufficient physical activity mitigated the adverse effects of extended sitting durations [19]. Additionally, cancer survivors with limited or no physical activity and prolonged sitting durations (> 8 h/day) faced up to a fivefold increase in risks of all-cause, cancer-specific, and non-cancer mortality [19].

The 2018 Physical Activity Guidelines for Americans [33] and the World Health Organization’s 2020 Global Guidelines on Physical Activity and Sedentary Behavior [18] have both highlighted the importance of minimizing sedentary behavior and replacing it with any form of physical activity to enhance health. This study supports these recommendations by providing empirical evidence of the interplay between physical activity and sedentary time in shaping mortality risks. The PASTBI score emerged as a valuable, unified metric that captures the combined impact of these behaviors and offers actionable insights for public health interventions.

Moreover, a prospective cohort study with 5836 Australian adults stated that participants in the lowest PASTBI category (characterized by lower non-occupational MVPA and higher sedentary time) had a 47% increased risk of all-cause mortality compared to those in the highest PASTBI category (characterized by higher non-occupational MVPA and lower sedentary time) [1.47 (1.21 − 1.79)] [20]. Consistent with these findings from cancer-free populations, the present study in cancer survivors demonstrated that those in the highest PASTBI category had approximately 50% lower risk of death from all causes, CVD, and cancer compared to those in the lowest PASTBI category. This suggests the potential of sufficient physical activity to mitigate the adverse effects of prolonged sitting, as Ekelund and colleagues’ findings [17].

An additional key finding of this study was that among individuals reporting no non-occupational MVPA, those with shorter sedentary time had a significantly lower risk of mortality. This pattern was evident in the analysis of PASTBI quartiles: participants in the lowest two quartiles (Q1 and Q2) reported no MVPA levels, but those in Q2 had reduced sedentary time, resulting in a higher PASTBI score and correspondingly lower mortality risk compared to Q1. These results underscore the utility of integrated behavioral measures like PASTBI, which capture subtle differences in movement patterns that may be missed when examining physical activity or sedentary behavior separately. Furthermore, this finding aligns with growing evidence that sedentary time independently influences mortality risk, highlighting the potential benefits of interventions specifically aimed at reducing sedentary behavior [34]. Clinically, this suggests that encouraging cancer survivors to interrupt prolonged sitting, such as by incorporating standing or light activity throughout the day, can yield substantial benefits, even for those unable or unwilling to participate in non-occupational MVPA programs.

This study is the first to examine the utility of the PASTBI score in assessing the combined impact of non-occupational MVPA and sedentary time on mortality risk among cancer survivors. Leveraging a large, nationally representative cohort and robust Cox regression models, our findings are both reliable and broadly generalizable. By distinguishing between sedentary time and physical inactivity, the study also offers practical insights for targeted interventions. Unlike traditional approaches that treat physical activity and sedentary behavior as separate exposures or categorize them using predefined thresholds, such as segmenting sitting time into hourly brackets or classifying activity based on guideline cutoffs, PASTBI provides a single, interpretable ratio that captures their behavioral balance. This integrated measure preserves more of the original data structure while streamlining model specification. Even when categorized, it offers a more efficient and intuitive representation of real-world movement patterns. As such, PASTBI may serve as a useful tool for guiding lifestyle recommendations, particularly in cancer survivors, some of whom may face challenges in engaging in structured physical activity.

However, this study is not without limitations. First, the self-reported nature of physical activity and sedentary time data may introduce recall bias and measurement error. Second, occupational physical activity was not included in the calculation of the PASTBI, which reflects an important limitation but also underscores the focus on modifiable and discretionary behaviors, such as leisure-time physical activity and sedentary habits. Third, although we adjusted for a wide range of covariates, data on cancer treatment and stage were not available in NHANES and therefore could not be included in the analysis. The time from cancer diagnosis to baseline assessment was used as a proxy to partially reflect disease severity or prognosis; however, this remains an imperfect substitute. Residual confounding from unmeasured or unknown factors cannot be fully excluded. In addition, the mean follow-up time of approximately 6 years may be insufficient to capture long-term mortality risks among cancer survivors. Finally, the observational design precludes definitive causal inferences, and further randomized trials are needed to validate the effectiveness of reducing sedentary behavior and increasing physical activity in improving survival outcomes for cancer survivors.

## Conclusion

In summary, this study offers new perspectives on the relationship between the ratio of non-occupational MVPA to sedentary behavior, as captured by the PASTBI score, and mortality risk in cancer survivors. The results demonstrated that a higher PASTBI score, indicating a more favorable balance between non-occupational MVPA and sedentary time, was strongly associated with reduced risks of all-cause, CVD, and cancer-related mortality. Additionally, even modest reductions in sedentary time have been shown to provide significant health benefits, independent of non-occupational MVPA levels. These results emphasized the dual importance of promoting physical activity and reducing sedentary behavior as complementary and actionable strategies to enhance survivorship outcomes in cancer survivors.

## Supplementary Information

Below is the link to the electronic supplementary material.

ESM 1 (DOCX 67.9 KB)

## Data Availability

Data can be available by visiting https://wwwn.cdc.gov/nchs/nhanes/ (accessed on December 10, 2024).
