# Supplementary material for: Association of Physical Activity and Sitting Time Balance Index with all-cause and cause-specific mortality among cancer survivors in the USA: a cohort study
Source: Support Care Cancer. 2025 Jul 2;33(7):653. doi: 10.1007/s00520-025-09709-x (PMC12222415; doi:10.1007/s00520-025-09709-x)
Supplement: Supplementary file 1 — (DOCX 67.9 KB) [file 520_2025_9709_MOESM1_ESM.docx]

**Online Resource**

**for**

**Association of Physical Activity and Sitting Time Balance Index with all-cause and cause-specific mortality among cancer survivors in the United States: a cohort study**

Yanxue Lian ^1, a, *^, Pincheng Luo ^1, a^

^1^ School of Medicine, University of Galway

^a^ Both authors contributed equally.

* Correspondence: [y.lian5@universityofgalway.ie](mailto:y.lian5@universityofgalway.ie)

**Table S1.** Baseline characteristics of cancer survivors by causes of mortality from NHANES 2007-2018.

**Table S2.** Sensitivity analysis considering the time from cancer diagnosis to baseline measurement.

**Table S3.** Sensitivity analysis excluding cancer survivors with less than 2 years of follow-up (461 of them were excluded).

**Table S4.** Sensitivity analysis after excluding those with BMI < 18.5 kg/m2 (50 of them were excluded).

**Table S5.** Sensitivity analysis after excluding those with CVD including angina, coronary heart disease, congestive heart failure, heart attack, and stroke (814 of them were excluded).

**Table S6.** Sensitivity analysis using Fine-Gray competing risk models on the associations between PASTBI and cause-specific mortality.

**Table S1.** Baseline characteristics of cancer survivors by causes of mortality from NHANES 2007-2018.

|  | **Total**  **(N = 3334)** | **All-cause** | | | **Cardiovascular** | | | **Malignant neoplasms** | | |
| --- | --- | --- | --- | --- | --- | --- | --- | --- | --- | --- |
|  |  | **Yes**  **(N = 849)** | **No**  **(N = 2485)** | ***P*-value** | **Yes**  **(N = 183)** | **No**  **(N = 3151)** | ***P*-value** | **Yes**  **(N = 297)** | **No**  **(N = 3037)** | ***P*-value** |
| **Age (yrs)** | 65.7 ±  13.9 | 73.6 ±  8.9 | 63.0 ±  14.3 | < 0.001 | 76.1 ±  7.3 | 65.1 ±  14.0 | <0.001 | 70.9 ±  9.6 | 65.2 ±  14.2 | <0.001 |
| **Age (yrs)** |  |  |  | <0.001 |  |  | <0.001 |  |  | <0.001 |
| <40 | 217  (6.51%) | 4  (0.47%) | 213  (8.57%) |  | 1  (0.55%) | 216  (6.85%) |  | 2  (0.67%) | 215  (7.08%) |  |
| 65>age≥40 | 1098  (32.93%) | 133  (15.67%) | 965  (38.83%) |  | 14  (7.65%) | 1084  (34.40%) |  | 71  (23.91%) | 1027  (33.82%) |  |
| ≥65 | 2019  (60.56%) | 712  (83.86%) | 1307  (52.60%) |  | 168  (91.80%) | 1851  (58.74%) |  | 224  (75.42%) | 1795  (59.10%) |  |
| **Gender** |  |  |  | <0.001 |  |  | <0.001 |  |  | <0.001 |
| Male | 1586  (47.57%) | 504  (59.36%) | 1082  (43.54%) |  | 115  (62.84%) | 1471  (46.68%) |  | 187  (62.96%) | 1399  (46.07%) |  |
| Female | 1748  (52.43%) | 345  (40.64%) | 1403  (56.46%) |  | 68  (37.16%) | 1680  (53.32%) |  | 110  (37.04%) | 1638  (53.93%) |  |
| **Race** |  |  |  | <0.001 |  |  | <0.001 |  |  | 0.154 |
| Mexican American | 231  (6.93%) | 33  (3.89%) | 198  (7.97%) |  | 4  (2.19%) | 227  (7.20%) |  | 17  (5.72%) | 214  (7.05%) |  |
| Other Hispanic | 225  (6.75%) | 29  (3.42%) | 196  (7.89%) |  | 4  (2.19%) | 221  (7.01%) |  | 13  (4.38%) | 212  (6.98%) |  |
| Non-Hispanic White | 2189  (65.66%) | 626  (73.73%) | 1563  (62.90%) |  | 149  (81.42%) | 2040  (64.74%) |  | 195  (65.66%) | 1994  (65.66%) |  |
| Non-Hispanic Black | 490  (14.70%) | 128  (15.08%) | 362  (14.57%) |  | 22  (12.02%) | 468  (14.85%) |  | 55  (18.52%) | 435  (14.32%) |  |
| Other Race | 199  (5.97%) | 33  (3.89%) | 166  (6.68%) |  | 4  (2.19%) | 195  (6.19%) |  | 17  (5.72%) | 182  (5.99%) |  |
| **Marital status** |  |  |  | <0.001 |  |  | <0.001 |  |  | 0.490 |
| Married | 1852  (55.55%) | 410  (48.29%) | 1442  (58.03%) |  | 81  (44.26%) | 1771  (56.20%) |  | 155  (52.19%) | 1697  (55.88%) |  |
| Widowed | 599  (17.97%) | 251  (29.56%) | 348  (14.00%) |  | 67  (36.61%) | 532  (16.88%) |  | 63  (21.21%) | 536  (17.65%) |  |
| Divorced | 445  (13.35%) | 106  (12.49%) | 339  (13.64%) |  | 18  (9.84%) | 427  (13.55%) |  | 45  (15.15%) | 400  (13.17%) |  |
| Separated | 105  (3.15%) | 21  (2.47%) | 84  (3.38%) |  | 6  (3.28%) | 99  (3.14%) |  | 7  (2.36%) | 98  (3.23%) |  |
| Never married | 223  (6.69%) | 46  (5.42%) | 177  (7.12%) |  | 8  (4.37%) | 215  (6.82%) |  | 19  (6.40%) | 204  (6.72%) |  |
| Living with partner | 110  (3.30%) | 15  (1.77%) | 95  (3.82%) |  | 3  (1.64%) | 107  (3.40%) |  | 8  (2.69%) | 102  (3.36%) |  |
| **Education** |  |  |  | <0.001 |  |  | 0.197 |  |  | <0.001 |
| Less than 9th grade | 309  (9.27%) | 117  (13.78%) | 192  (7.73%) |  | 23  (12.57%) | 286  (9.08%) |  | 40  (13.47%) | 269  (8.86%) |  |
| 9-11th grade | 411  (12.33%) | 150  (17.67%) | 261  (10.50%) |  | 28  (15.30%) | 383  (12.15%) |  | 50  (16.84%) | 361  (11.89%) |  |
| High school graduate | 730  (21.90%) | 196  (23.09%) | 534  (21.49%) |  | 43  (23.50%) | 687  (21.80%) |  | 73  (24.58%) | 657  (21.63%) |  |
| Some college or AA degree | 1009  (30.26%) | 210  (24.73%) | 799  (32.15%) |  | 47  (25.68%) | 962  (30.53%) |  | 77  (25.93%) | 932  (30.69%) |  |
| College graduate or above | 875  (26.24%) | 176  (20.73%) | 699  (28.13%) |  | 42  (22.95%) | 833  (26.44%) |  | 57  (19.19%) | 818  (26.93%) |  |
| **PIR** |  |  |  | <0.001 |  |  | 0.051 |  |  | <0.001 |
| <1 | 479  (14.37%) | 136  (16.02%) | 343  (13.80%) |  | 27  (14.75%) | 452  (14.34%) |  | 62  (20.88%) | 417  (13.73%) |  |
| 2>PIR≥1 | 813  (24.39%) | 274  (32.27%) | 539  (21.69%) |  | 52  (28.42%) | 761  (24.15%) |  | 90  (30.30%) | 723  (23.81%) |  |
| 3> PIR≥2 | 802  (24.06%) | 217  (25.56%) | 585  (23.54%) |  | 53  (28.96%) | 749  (23.77%) |  | 68  (22.90%) | 734  (24.17%) |  |
| ≥3 | 1240  (37.19%) | 222  (26.15%) | 1018  (40.97%) |  | 51  (27.87%) | 1189  (37.73%) |  | 77  (25.93%) | 1163  (38.29%) |  |
| **BMI (kg/m2)** | 29.1 ±  6.4 | 28.2 ±  6.1 | 29.4 ±  6.5 | < 0.001 | 29.1 ±  5.7 | 29.1 ±  6.4 | 0.958 | 28.3 ±  5.7 | 29.2 ±  6.5 | 0.013 |
| **BMI (kg/m2)** |  |  |  | <0.001 |  |  | 0.039 |  |  | 0.173 |
| <18.5 | 50  (1.50%) | 22  (2.59%) | 28  (1.13%) |  | 1  (0.55%) | 49  (1.56%) |  | 7  (2.36%) | 43  (1.42%) |  |
| 25>BMI≥18.5 | 789  (23.67%) | 215  (25.32%) | 574  (23.10%) |  | 37  (20.22%) | 752  (23.87%) |  | 69  (23.23%) | 720  (23.71%) |  |
| 30> BMI≥25 | 1298  (38.93%) | 364  (42.87%) | 934  (37.59%) |  | 89  (48.63%) | 1209  (38.37%) |  | 128  (43.10%) | 1170  (38.52%) |  |
| ≥30 | 1197 (35.90%) | 248 (29.21%) | 949  (38.19%) |  | 56  (30.60%) | 1141  (36.21%) |  | 93  (31.31%) | 1104  (36.35%) |  |
| **Angina** |  |  |  | <0.001 |  |  | <0.001 |  |  | 0.339 |
| Yes | 174  (5.22%) | 65  (7.66%) | 109  (4.39%) |  | 25  (13.66%) | 149  (4.73%) |  | 12  (4.04%) | 162  (5.33%) |  |
| No | 3160  (94.78%) | 784  (92.34%) | 2376  (95.61%) |  | 158  (86.34%) | 3002  (95.27%) |  | 285  (95.96%) | 2875  (94.67%) |  |
| **Coronary Heart Disease** |  |  |  | <0.001 |  |  | <0.001 |  |  | 0.232 |
| Yes | 317  (9.51%) | 131  (15.43%) | 186  (7.48%) |  | 43  (23.50%) | 274  (8.70%) |  | 34  (11.45%) | 283  (9.32%) |  |
| No | 3017  (90.49%) | 718  (84.57%) | 2299  (92.52%) |  | 140  (76.50%) | 2877  (91.30%) |  | 263  (88.55%) | 2754  (90.68%) |  |
| **Congestive Heart Failure** |  |  |  | <0.001 |  |  | <0.001 |  |  | 0.093 |
| Yes | 235  (7.05%) | 117  (13.78%) | 118  (4.75%) |  | 36  (19.67%) | 199  (6.32%) |  | 28  (9.43%) | 207  (6.82%) |  |
| No | 3099  (92.95%) | 732  (86.22%) | 2367  (95.25%) |  | 147  (80.33%) | 2952  (93.68%) |  | 269 (90.57%) | 2830  (93.18%) |  |
| **Diabetes** |  |  |  | <0.001 |  |  | <0.001 |  |  | 0.020 |
| Yes | 920  (27.59%) | 299 (35.22%) | 621  (24.99%) |  | 74  (40.44%) | 846  (26.85%) |  | 99  (33.33%) | 821  (27.03%) |  |
| No | 2414  (72.41%) | 550  (64.78%) | 1864  (75.01%) |  | 109  (59.56%) | 2305  (73.15%) |  | 198  (66.67%) | 2216  (72.97%) |  |
| **Heart attack** |  |  |  | <0.001 |  |  | <0.001 |  |  | 0.002 |
| Yes | 327  (9.81%) | 145  (17.08%) | 182  (7.32%) |  | 42  (22.95%) | 285  (9.04%) |  | 44  (14.81%) | 283  (9.32%) |  |
| No | 3007  (90.19%) | 704  (82.92%) | 2303  (92.68%) |  | 141  (77.05%) | 2866  (90.96%) |  | 253  (85.19%) | 2754  (90.68%) |  |
| **Hypertension** |  |  |  | <0.001 |  |  | <0.001 |  |  | 0.137 |
| Yes | 2591  (77.71%) | 727  (85.63%) | 1864  (75.01%) |  | 168  (91.80%) | 2423  (76.90%) |  | 241  (81.14%) | 2350  (77.38%) |  |
| No | 743  (22.29%) | 122  (14.37%) | 621  (24.99%) |  | 15  (8.20%) | 728  (23.10%) |  | 56  (18.86%) | 687  (22.62%) |  |
| **Stroke** |  |  |  | <0.001 |  |  | <0.001 |  |  | 0.012 |
| Yes | 295  (8.85%) | 131  (15.43%) | 164  (6.60%) |  | 38  (20.77%) | 257  (8.16%) |  | 38  (12.79%) | 257  (8.46%) |  |
| No | 3039  (91.15%) | 718  (84.57%) | 2321  (93.40%) |  | 145  (79.23%) | 2894 (91.84%) |  | 259  (87.21%) | 2780  (91.54%) |  |
| **Alcohol consumption** |  |  |  | <0.001 |  |  | <0.001 |  |  | <0.001 |
| 1 | 924  (27.71%) | 203  (23.91%) | 721  (29.01%) |  | 53  (28.96%) | 871  (27.64%) |  | 59  (19.87%) | 865  (28.48%) |  |
| 2-14 | 860  (25.79%) | 136  (16.02%) | 724  (29.13%) |  | 23  (12.57%) | 837  (26.56%) |  | 63  (21.21%) | 797  (26.24%) |  |
| ≥15 | 5  (0.15%) | 0  (0%) | 5  (0.20%) |  | 0  (0%) | 5  (0.16%) |  | 0  (0%) | 5  (0.16%) |  |
| Missing | 1545  (46.34%) | 510  (60.07%) | 1035  (41.65%) |  | 107  (58.47%) | 1438  (45.64%) |  | 175  (58.92%) | 1370  (45.11%) |  |
| **Smoking status** |  |  |  | <0.001 |  |  | 0.042 |  |  | 0.003 |
| Never smoker | 1522  (45.65%) | 315  (37.10%) | 1207  (48.57%) |  | 76  (41.53%) | 1446  (45.89%) |  | 109  (36.70%) | 1413  (46.53%) |  |
| Former smoker | 1290  (38.69%) | 412  (48.53%) | 878  (35.33%) |  | 86  (46.99%) | 1204  (38.21%) |  | 139  (46.80%) | 1151  (37.90%) |  |
| Current smoker | 522  (15.66%) | 122  (14.37%) | 400 (16.10%) |  | 21  (11.48%) | 501  (15.90%) |  | 49  (16.50%) | 473  (15.57%) |  |
| **Sedentary time**  **(hours/weekly)** | 44.7 ±  23.0 | 48.1 ±  22.6 | 43.6 ±  23.0 | <0.001 | 48.3 ±  21.3 | 44.5 ±  23.1 | 0.03 | 45.9 ±  21.5 | 44.6 ±  23.1 | 0.37 |
| **PASTBI** | 7.2 ±  25.5 | 3.6 ±  11.6 | 8.5 ±  28.6 | <0.001 | 3.7 ±  11.7 | 7.5 ±  26.0 | 0.054 | 4.3 ±  11.2 | 7.5 ±  26.4 | 0.035 |
| **Total physical activity (Minutes/weekly)** | 184.71 ±  360.45 | 99.87 ±  253.11 | 213.70 ±  386.20 | <0.001 | 105.04 ±  270.76 | 189.34 ±  364.48 | 0.002 | 114.37 ±  260.03 | 191.59 ±  368.11 | <0.001 |
| **Time from cancer diagnosis to baseline measurement (years)** | 10.09 ± 10.53 | 9.68 ±  11.83 | 10.23 ±  10.05 | 0.19 | 10.70 ±  11.99 | 10.06 ±  10.44 | 0.42 | 7.53 ±  11.16 | 10.34 ±  10.44 | <0.001 |
| **Months of follow-up** | 72.02 ±  42.01 | 53.09 ±  36.50 | 78.49 ±  41.82 | <0.001 | 54.91 ±  35.54 | 73.01 ± 42.15 | <0.001 | 46.80 ±  36.92 | 74.49 ±  41.67 | <0.001 |

Continuous variables are presented as means ± standard deviation (SD), and while categorical variables are presented as frequencies (%).

**Abbreviations:** PASTBI, Physical Activity and Sitting Time Balance Index; BMI, body mass index; PIR, poverty-income ratio

**Table S2.** Sensitivity analysis considering the time from cancer diagnosis to baseline measurement.

| **PASTBI quartiles** | **No. of Events (%)** | **Full adjusted HR (95%CI), *P*-value** |
| --- | --- | --- |
| **All-cause mortality** | **849 (25.46%)** |  |
| Quartile 1 | 317 (39.53%) | Reference |
| Quartile 2 | 249 (29.12%) | 0.62 (0.49; 0.78) |
| Quartile 3 | 159 (18.91%) | 0.38 (0.30; 0.49) |
| Quartile 4 | 124 (14.83%) | 0.38 (0.29; 0.50) |
| *P* for trend |  | < 0.0001 |
| **CVD mortality** | **183 (5.49%)** |  |
| Quartile 1 | 69 (8.60%) | Reference |
| Quartile 2 | 48 (5.61%) | 0.54 (0.35; 0.84) |
| Quartile 3 | 40 (4.76%) | 0.49 (0.30; 0.81) |
| Quartile 4 | 26 (3.11%) | 0.45 (0.24; 0.88) |
| *P* for trend |  | < 0.01 |
| **Cancer mortality** | **297 (8.91%)** |  |
| Quartile 1 | 98 (12.22%) | Reference |
| Quartile 2 | 93 (10.88%) | 0.74 (0.53; 1.04) |
| Quartile 3 | 56 (6.66%) | 0.44 (0.29; 0.67) |
| Quartile 4 | 50 (5.98%) | 0.44 (0.27; 0.72) |
| *P* for trend |  | 0.0004 |

Adjusted for all covariates in the primary analysis (including age, gender, race, marital status, education level, PIR, BMI, smoking status, and alcohol consumption, hypertension, diabetes, angina, coronary heart disease, heart attack, congestive heart failure, and stroke) and the time from cancer diagnosis to baseline measurement. **Abbreviations:** PASTBI, Physical Activity and Sitting Time Balance Index; CVD, cardiovascular diseases; BMI, body mass index; PIR, poverty-income ratio.

**Table S3.** Sensitivity analysis excluding cancer survivors with less than 2 years of follow-up (461 of them were excluded).

| **PASTBI quartiles** | **No. of Events (%)** | **Full adjusted HR (95%CI), *P*-value** |
| --- | --- | --- |
| **All-cause mortality** | 631 (21.96%) |  |
| Quartile 1 | 221 (34.42%) | Reference |
| Quartile 2 | 180 (25.10%) | 0.63 (0.48; 0.83) |
| Quartile 3 | 125 (16.58%) | 0.41 (0.30; 0.54) |
| Quartile 4 | 105 (16.58%) | 0.45 (0.33; 0.61) |
| *P* for trend |  | < 0.0001 |
| **CVD mortality** | **141 (4.91%)** |  |
| Quartile 1 | 46 (7.17%) | Reference |
| Quartile 2 | 38 (5.30%) | 0.64 (0.40; 1.04) |
| Quartile 3 | 34 (4.51%) | 0.56 (0.33; 0.95) |
| Quartile 4 | 23 (3.03%) | 0.57 (0.30; 1.15) |
| *P* for trend |  | 0.04 |
| **Cancer mortality** | **192 (6.68%)** |  |
| Quartile 1 | 58 (9.03%) | Reference |
| Quartile 2 | 58 (8.09%) | 0.82 (0.54; 1.25) |
| Quartile 3 | 38 (5.04%) | 0.48 (0.28; 0.81) |
| Quartile 4 | 38 (5.00%) | 0.54 (0.33; 0.88) |
| *P* for trend |  | 0.004 |

Adjusted for all covariates in the primary analysis (including age, gender, race, marital status, education level, PIR, BMI, smoking status, and alcohol consumption, hypertension, diabetes, angina, coronary heart disease, heart attack, congestive heart failure, and stroke).

**Abbreviations**: PASTBI, Physical Activity and Sitting Time Balance Index; CVD, cardiovascular diseases; BMI, body mass index; PIR, poverty-income ratio.

**Table S4.** Sensitivity analysis after excluding those with BMI < 18.5 kg/m2 (50 of them were excluded).

| **PASTBI quartiles** | **No. of Events (%)** | **Full adjusted HR (95%CI), *P*-value** |
| --- | --- | --- |
| **All-cause mortality** | **827 (25.18%)** |  |
| Quartile 1 | 311 (39.42%) | Reference |
| Quartile 2 | 239 (28.49%) | 0.61 (0.48; 0.78) |
| Quartile 3 | 155 (18.74%) | 0.39 (0.30; 0.50) |
| Quartile 4 | 122 (14.72%) | 0.38 (0.29; 0.50) |
| *P* for trend |  | < 0.0001 |
| **CVD mortality** | **182 (5.54%)** |  |
| Quartile 1 | 69 (8.75%) | Reference |
| Quartile 2 | 47 (5.60%) | 0.54 (0.35; 0.83) |
| Quartile 3 | 40 (4.84%) | 0.49 (0.30; 0.81) |
| Quartile 4 | 26 (3.14%) | 0.45 (0.24; 0.86) |
| *P* for trend |  | 0.009 |
| **Cancer mortality** | **290 (8.83%)** |  |
| Quartile 1 | 96 (12.17%) | Reference |
| Quartile 2 | 90 (10.73%) | 0.76 (0.53; 1.08) |
| Quartile 3 | 55 (6.65%) | 0.45 (0.29; 0.69) |
| Quartile 4 | 49 (5.91%) | 0.46 (0.27; 0.76) |
| *P* for trend |  | 0.0005 |

Adjusted for all covariates in the primary analysis (including age, gender, race, marital status, education level, PIR, BMI, smoking status, and alcohol consumption, hypertension, diabetes, angina, coronary heart disease, heart attack, congestive heart failure, and stroke).

**Abbreviations:** PASTBI, Physical Activity and Sitting Time Balance Index; CVD, cardiovascular diseases; BMI, body mass index; PIR, poverty-income ratio.

**Table S5.** Sensitivity analysis after excluding those with CVD including angina, coronary heart disease, congestive heart failure, heart attack, and stroke (814 of them were excluded).

| **PASTBI quartiles** | **No. of Events (%)** | **Full adjusted HR (95%CI), *P*-value** |
| --- | --- | --- |
| **All-cause mortality** | **515 (20.44%)** |  |
| Quartile 1 | 183 (34.40%) | Reference |
| Quartile 2 | 148 (23.87%) | 0.57 (0.43; 0.76) |
| Quartile 3 | 99 (14.82%) | 0.36 (0.26; 0.50) |
| Quartile 4 | 85 (12.14%) | 0.35 (0.24; 0.51) |
| *P* for trend |  | < 0.0001 |
| **CVD mortality** | **76 (3.02%)** |  |
| Quartile 1 | 27 (5.08%) | Reference |
| Quartile 2 | 19 (3.06%) | 0.56 (0.25; 1.26) |
| Quartile 3 | 21 (3.14%) | 0.53 (0.22; 1.32) |
| Quartile 4 | 9 (1.29%) | 0.30 (0.10; 0.95) |
| *P* for trend |  | 0.04 |
| **Cancer mortality** | **205 (8.13%)** |  |
| Quartile 1 | 65 (12.22%) | Reference |
| Quartile 2 | 61 (9.84%) | 0.64 (0.40; 1.02) |
| Quartile 3 | 37 (5.54%) | 0.38 (0.21; 0.66) |
| Quartile 4 | 42 (6.00%) | 0.44 (0.24; 0.84) |
| *P* for trend |  | < 0.01 |

Adjusted for all covariates in the primary analysis (including age, gender, race, marital status, education level, PIR, BMI, smoking status, and alcohol consumption, hypertension, diabetes).

**Abbreviations:** PASTBI, Physical Activity and Sitting Time Balance Index; CVD, cardiovascular diseases; BMI, body mass index; PIR, poverty-income ratio.

**Table S6.** Sensitivity analysis using Fine-Gray competing risk models on the associations between PASTBI and cause-specific mortality.

| **PASTBI quartiles** | **Cancer mortality** | **CVD mortality** |
| --- | --- | --- |
|  | **Subdistribution HR (95% CI)** | **Subdistribution HR (95% CI)** |
| Quartile 1 | Reference | Reference |
| Quartile 2 | 0.91 (0.66; 1.26) | 0.95 (0.61; 1.47) |
| Quartile 3 | 0.56 (0.39; 0.81) | 0.69 (0.42; 1.14) |
| Quartile 4 | 0.51 (0.35; 0.76) | 0.53 (0.31; 0.90) |
| *P* for trend | 0.0001 | 0.01 |

Adjusted for age, gender, race, marital status, education level, PIR, hypertension, diabetes.

**Abbreviations:** PASTBI, Physical Activity and Sitting Time Balance Index; CVD, cardiovascular diseases; PIR, poverty-income ratio.
